# Introduction to the Special Issue on *Clostridioides difficile* Infection, Second Edition

**DOI:** 10.3390/antibiotics13070607

**Published:** 2024-06-29

**Authors:** Guido Granata

**Affiliations:** Clinical and Research Department for Infectious Diseases, National Institute for Infectious Diseases L. Spallanzani, IRCCS, 00149 Rome, Italy; guido.granata@inmi.it

*Clostridioides difficile* (CD) is a Gram-positive, anaerobic bacterium that is one of the most common causes of infective diarrhoea worldwide [1].

Among hospitalized patients, *Clostridioides difficile* infection (CDI) leads to increased morbidity, mortality, and extended hospital stays. Despite research efforts and progress made regarding the epidemiology and clinical management of CDI in the last decade, several critical aspects of this complex disease remain unclear. Firstly, the clinical spectrum of CDI is wide-ranging, from asymptomatic carriage and mild diarrhoea to severe colitis, toxic megacolon, and fatality [1,2]. The mortality rates associated with CDI vary considerably across studies, ranging from less than 2% to 17% [2]. 

The identification of CD carriers at high risk of developing infection and CDI patients at high risk of developing severe CDI and experiencing recurrence remains a significant challenge [3].

Another concern is the intra-hospital and community spread of CD [4]. Understanding the transmission routes is crucial for the development of targeted interventions to reduce the spread of CDI. Antimicrobial stewardship and infection control programmes may help to prevent CDI, even during the ongoing SARS-CoV-2 pandemic [4].

The molecular pathogenesis of CDI remains uncertain. Further research is required to elucidate the interactions between CD, the gut microbiota, host immunity, and the specific roles of CD toxin A, toxin B, and binary toxin [5,6]. 

Finally, CDI recurrences present a significant challenge, increasing hospitalization costs and morbidity and mortality rates [3]. While oral vancomycin or fidaxomicin represent the recommended first-line antimicrobial therapies, further study is required to confirm the efficacy and safety of innovative non-antimicrobial approaches, including monoclonal anti-toxin antibodies, fecal microbiota transplantation, vaccines, and phage therapy [1,7].

The Second Edition of the Special Issue *Clostridioides difficile Infection* includes seven full research articles, two review articles, and one perspective and one communication article. These contributions aim to add clarity on the open issues surrounding this topic.

Among the contributions, the work by Stoian, M. et al. examined the correlation between COVID-19 and CDI in the intensive care unit [8]. Of interest, the authors identified immuno-modulator or steroid treatment, antibiotic administration, and proton pump inhibitor treatment as significant risk factors for CDI coinfection among COVID-19 patients admitted to intensive care, and reported an increased mortality rate among these patients [8].

The study by Lis L. et al. aimed to identify the clinical determinants predicting CDI among the subgroup of hospitalized patients with chronic kidney disease [9]. The results confirmed that serum albumin has a protective effect against CDI severity. The multivariate analysis showed that the stage of chronic kidney disease and the length of antibiotic use increased the risk of CDI, whereas a lower Norton scale score had a protective impact [9].

Regarding CDI treatment in specific subgroups of high-risk patients, Giacobbe, D. R. et al. discussed the updated evidence on the efficacy of fidaxomicin for the treatment of either the first CDI episode or recurrent CDI. The authors reported evidence supporting the use of fidaxomicin despite its high cost. According to the authors, risk models for recurrent CDI should be used to select patients for fidaxomicin treatment [10].

Future studies should focus on identifying high-risk groups for recurrent CDI. At present, bezlotoxumab may be considered in specific, high-risk patients with immunosuppression. Granata, G. et al. collected the available evidence on bezlotoxumab for preventing recurrent CDI during a first CDI episode [11]. Their findings support the administration of bezlotoxumab in patients with a primary CDI episode, despite the high cost. According to the authors, it is likely that the future guidance may change from “administer bezlotoxumab only in high-risk patients” to “consider bezlotoxumab even for a primary CDI episode, in view of the patient benefits and the cost-effectiveness of reducing expensive recurrent CDI episodes” [11].

This Special Issue presents a compendium of multidisciplinary research on CDI. The collected works serve as a comprehensive resource for scholars engaged in the field of CDI, and the Guest Editor is grateful for the interest and contributions that have been received.
